# Knowledge and utilization of computer among health workers in Addis Ababa hospitals, Ethiopia: computer literacy in the health sector

**DOI:** 10.1186/1756-0500-6-106

**Published:** 2013-03-20

**Authors:** Ebrahim Mohammed, Gashaw Andargie, Solomon Meseret, Eshetu Girma

**Affiliations:** 1 Samara University, Samara, Ethiopia; 2 School of Public Health, College of Medicine and Health Sciences, University of Gondar, Gondar, Ethiopia; 3 Department of Health Education and Behavioral Sciences, Jimma University, Jimma, Ethiopia

**Keywords:** Computer, Knowledge, Utilization, Health care, Health workers, Computer literacy

## Abstract

**Background:**

Incorporation of information technology advancements in healthcare has gained wide acceptance in the last two decades. Developed countries have successfully incorporated information technology advancements in their healthcare system thus, improving healthcare. However, only a limited application of information technology advancements is seen in developing countries in their healthcare system. Hence, this study was aimed at assessing knowledge and utilization of computer among health workers in Addis Ababa hospitals.

**Methods:**

A quantitative cross-sectional study was conducted among 304 health workers who were selected using stratified sampling technique from all governmental hospitals in Addis Ababa. Data was collected from April 15 to April 30, 2010 using a structured, self-administered, and pre-tested questionnaire from five government hospitals in Addis Ababa. The data was entered into Epi Info version 3.5.1 and exported to SPSS version 16. Analysis was done using multinomial logistic regression technique.

**Results:**

A total of 270 participants, age ranging from 21 to 60 years responded to the survey (88.8% response rate). A total of 91 (33.7%) respondents had an adequate knowledge of computers while 108 (40.0%) had fair knowledge and 71(26.3%) of the respondents showed inadequate knowledge. A total of 38(14.1%) were adequately utilizing computers, 14(5.2%) demonstrated average or fair utilization and majority of the respondents 218(80.7%) inadequately utilized computers. Significant predictor variables were average monthly income, job satisfaction index and own computer possession.

**Conclusions:**

Computer knowledge and utilization habit of health workers were found to be very low. Increasing accessibility to computers and delivering training on the use of computers for workers will increases the knowledge and utilization of computers. This will facilitate the rate of diffusion of the technology to the health sector. Hence, programs targeted at enhancing knowledge and skill of computer use and increasing access to computer should be designed. The association between computer knowledge/skill and health care delivery competence should be studied.

## Background

Since the advent of computers, there has been significant interests in the collection, storage, retrieval, and analysis of a wide range of information in all spheres of socioeconomic development endeavor [1]. In the health sector, advances in information and computer technology in the last quarter of the twentieth century has led to the ability to more accurately profile individual health risk, to understand better basic physiologic and pathologic processes and diagnosis through new imaging and scanning technologies. Such technological development, however, demands an increased responsibility of practitioners, managers, and policy-makers to assess appropriateness of new technologies [2].

Information technology is at the heart of modern healthcare systems and services and can distribute information world-wide. National and international e-health initiatives are challenged by deep-rooted problems and lack of infrastructures like access to computers. Many remote villages that lack easy access to hospitals and advanced medical facilities are now being educated through telemedicine and digitalized health information. This initiative is helping millions of citizens improve their daily lives [3].

A survey in Ethiopia indicated that 46% of the schools/colleges, 41% of the public institutions use computers for administration. Among different institutions, only 28% of the health facilities which was very low as compared to other institution used computer for different purposes [4]. In contrary, there was high knowledge and use of computers among dental sciences faculty, at the University of Lagos, Nigeria [5].

A study in a Nigerian University Teaching hospital in 2004 found that 18.9% of health professionals demonstrated a good knowledge of computers while 58.8% had average knowledge. Only 22.3% showed poor knowledge. Among these study participants, only 39.9% demonstrated good utilization habits, while in 33.8% utilization habits were average [6]. Another study conducted among clinical year medical students of Ahmadu Bello University, in Nigeria showed that 50.6% of the participants had knowledge about computer technology and its use while 49.4% did not know much about computers and their use [7]. A survey conducted in northern Ethiopia showed that computer illiteracy rate was 89.41%. From the overall workforce surveyed in Ethiopia, computer trained workforce accounted only for 0.17% [8].

Previous studies identified the following as factors affecting computer utilization: computers, training on the use of computers and age [1,6,8-11].

Ethiopia is in a position to implement the Health management information system (HMIS). This involves computer application starting from entering patient records to the use telemedicine to educate healthcare providers on the cases that are beyond the capacity of Ethiopian Hospitals. Little is known about the knowledge and utilization patterns of computer, and there are no published reports on the knowledge, utilization and factors affecting utilization of computer among health workers in Ethiopia. To fill the gap, it was important to assess knowledge of health workers that might predict their acceptance and mode of use of computer systems. These include how much they know about computers. How many health workers currently use computers and factor affecting utilization habit of computer?

## Methods

Institution based quantitative cross-sectional study design was used to assess knowledge, utilization and factors affecting knowledge and utilization of computer among health workers. The study was conducted in Addis Ababa, the capital city of Ethiopia. It is the largest city in Ethiopia, with a population of 3,384,569 according to the 2008 population census [12]. There are five governmental hospitals which are governed by Addis Ababa administrative health bureau; namely, Ras Desta, Gandi, Minilik, Yekatit 12, and Zewditu Hospital. The study was carried out from April to June, 2010.

Sample of 304 health workers in the five hospitals were included in the study. Only those health workers who have worked in the study area at least for 6 months were included in the study. The sample size was calculated using single population proportion formula by assuming proportion of people with adequate knowledge of computer 0.59 [6] , 95% confidence interval, 0.05 margin of error and 10% contingency for non-response. Final correction formula was also used. The study participants were selected using simple random sampling technique with proportional allocation among the five hospitals. There were a total of 1072 total health workers in the five hospitals. Based on the proportion of health workers in each hospital, 33 health workers from Rass Desta, 35 from Gandi, 70 from Minilik, 77 from Yekatit twelve and 55 from Zewditu were included. Lists of health workers were prepared for each hospital as sampling frames. A pre-tested self administered questionnaire was used to collect data on socio-demographic characteristics, computer knowledge and utilization of health workers was adopted from previous study [9]. The questionnaire was prepared in English language. The data collection was facilitated by 5 information technology diploma holders and supervision was done by the principal investigator.

In this study health workers were those employees with at least a diploma certificate on health profession. Computer knowledge was defined as a basic understanding of computer concepts. It involves knowing hard and soft ware, what computer virus mean how to manage files, and knowing how to use basic computer applications like computer network, information highway and internet. A total of 20 questions were used to assess the knowledge of computer. Based on the cut of value from the Nigerian study in 2004 on the same topic it was classified as follows for both knowledge and utilization of computer among health workers. (1) Adequate computer knowledge: those study subjects who scored greater than 80% of knowledge questions, (2) Fair computer knowledge: those study subjects who scored 60% to 79% of knowledge questions and (3) inadequate computer knowledge: those study subjects who scored less than 60% of knowledge questions. Utilization of computer is the basic skill and use of computer and internet, manage files, storing, retrieve, analyzing and presenting the data on hand. A total of 15 questions were used to assess health worker’s computer utilization habits. (1) Adequate computer utilization: those study subjects who scored greater than 60% of utilization questions). (2) Fair computer utilization: those study subjects who scored 50% to 59% of utilization questions. (3) Inadequate computer utilization: those study subjects who scored less than 50% of utilization question.

Data was entered using Epi Info for double data entry. Then it was exported to SPSS package version 16 for analysis. Frequencies and cross tabulations were used for the descriptive analysis of the data. Associations between participant’s characteristics and knowledge and utilization of computer were analyzed using multinomial logistic regression. Ethical clearance committee of the University of Gondar College of Medicine and Health science through School of Public Health approved this study. Data was collected after getting permission and clearance from ethical clearance committee of Addis Ababa administrative health bureau Written consent was obtained from each respondent.

## Results

### Socio- demographic characteristics

The response rate was 270 (88.8%). Female to male ratio was 1.23: 1 (149 females and 121 males). The median age of the respondents was 29 years. Never married and ever married study participants were 114(42.2%) and 156(57.8%) respectively. One hundred and fifty eight (58.5%) of the respondents were Orthodox. One hundred and forty two (52.6%) of them were paid less than or equal to 1500 ETHB (1 USD = 18.4 ETHB) monthly salary. Regarding job satisfaction of the respondents, 49.4%.of respondents were satisfied with their work. One hundred and forty (51.9%) of the respondents were nurses, majority of whom have either a bachelors degree or a diploma (89.3%) (Table 1).

**Table 1:** Socio-demographic characteristics of respondents in Addis Ababa administrative hospitals Addis Ababa Ethiopia, June 2010

**Variables**	**Hospitals**					**Total, n=270(%)**
	**Ras Desta, n=33(%)**	**Gandi, n=35(%)**	**Zewditu, n=55(%)**	**Yekatit 12, n=77(%)**	**Minilik, n=70(%)**	
**Sex**						
Females	15(5.6)	28(10.4)	39(14.4)	44(16.3)	23(8.5)	149(55.2)
Males	18(6.7)	27(10.0)	31(11.5)	33(12.2)	12(4.4)	121(44.8)
**Age**						
≤ 25	4(1.5)	8(3.0)	15(5.6)	21(7.8)	10(3.7)	58(21.5)
26-29	13(4.8)	10(3.7)	25(9.3)	18(6.7)	15(5.6)	81(30.0)
≥ 30	16(5.9)	17(6.3)	30(11.1)	38(14.1)	30(11.1)	131(48.5)
**Marital status**						
Ever married	18(6.7)	19(7.0)	37(13.7)	49(18.1)	33(12.2)	156(57.8)
Never married	15(5.6)	16(5.9)	33(12.2)	28(10.4)	22(8.1)	114(42.2)
**Religion**						
Orthodox	15(5.6)	16(5.9)	50(18.5)	49(18.1)	28(10.4)	158(58.5)
Protestant	10(3.7)	14(5.2)	12(4.4)	20(7.4)	16(5.9)	72(26.7)
Muslim	8(3.0)	5(1.9)	8(3.0)	8(3.0)	11(4.1)	40(14.8)
**Monthly income**						
Low	16(5.9)	23(8.5)	29(10.7)	36(13.3)	38(14.1)	142(52.6)
High	17(6.3)	12(4.4)	26(9.6)	41(15.2)	32(11.9)	128(47.4)
**Field of study**						
Nurse	8(3.0)	22(8.1)	39(14.4)	47(17.4)	24(8.9)	140(51.9)
Lab and pharmacy	13(4.8)	8(3.0)	10(3.7)	12(4.4)	17(6.3)	60(22.2)
MD and HO	6(2.2)	3(1.1)	13(4.8)	9(3.3)	6(2.2)	37(13.7)
Others	6(2.2)	2(.7)	8(3.0)	9(3.3)	8(3.0)	33(12.2)
**Educational level**						
BSc and diploma	28(10.4)	32(11.9)	50(18.5)	72(26.7)	59(21.9)	241(89.3)
Above BSc	5(1.9)	3(1.1)	5(1.9)	5(1.9)	11(4.1)	29(10.7)

*MD *Medical doctor; *HO *Health officer & others include: environmental health, dentistry, physiotherapy & radiography.

### Computer possession, training and job satisfaction

Hundred thirty four (49.6%) respondents had computer training before. Majority of the health workers 203 (75.2%) had no own computer. A total of 133 (49.3%) them were satisfied with their work (Table 2).

**Table 2:** Job satisfaction, training received and computer possession among the respondents in Addis Ababa administrative hospitals Addis Ababa, Ethiopia, June 2010

**Variables**	**Hospitals**					**Total (%)**
	**Ras Desta(%)**	**Gandi (%)**	**Zewditu (%)**	**Yekatit 12 (%)**	**Minilik (%)**	
**Job satisfaction**						
Satisfied	11(4.1)	20(7.4)	32(11.9)	37(13.7)	33(12.2)	133(49.3)
Poorly satisfied	17(6.3)	9(3.3)	28(10.4)	27(10.0)	12(4.4)	93(34.4)
Very satisfied	5(1.9)	6(2.2)	10(3.7)	13(4.8)	10(3.7)	44(16.3)
**computer training**						
Trained	20(7.4)	20(7.4)	30(11.1)	41(15.2)	23(8.5)	134(49.6)
Not trained	13(4.8)	15(5.6)	40(14.8)	36(13.3)	32(11.9)	136(50.4)
**Owned computer**						
Owed	11(4.1)	10(3.7)	15(5.6)	21(7.8)	10(3.7)	67(24.8)
Not	22(8.1)	25(9.3)	55(20.4)	56(20.7)	45(16.7)	203(75.2)

### Knowledge on computer

A total of 91(33.7%) respondents demonstrated adequate knowledge of computers, 108 (40.0%) of them had fair knowledge while 71 (26.3%) showed inadequate knowledge. Results from the multinomial logistic regression analysis indicated that, those who never married were more knowledgeable [AOR- 3.05, 95% CI (1.11, 8.39)] than ever married individuals. People with less income were less knowledgeable [AOR- 0.26, 95% CI (0.11, 0.65)]. Regarding the job satisfaction of the respondents, respondents in the satisfied category had statistically significant higher knowledge (AOR- 2.67, 95% CI (1.12, 6.37)]. People with training on computers were more likely to be knowledgeable [AOR- 2.81, 95% CI (1.22, 6.43)] than without training Computer possession was also statistically associated (AOR- 6.35, 95% CI (2.00, 20.18)] with having high knowledge on computers (Table 3). Those people whose ages were between 25–29 years were more likely [AOR- 2.54, 95% CI (1.04, 6.22)] to have fair knowledge than people with more than 30 years age. And respondents with less income were less likely [AOR- 0.34, 95% CI (0.15, 0.77)] to have fair knowledge than high income groups (Table 4).

**Table 3:** factors associated with adequate knowledge of computer among health workers at Addis Ababa administrative hospitals Ethiopia, June 2010

**Predictor variable**	**Knowledge**		**COR (95% CI)**	**AOR (95% CI)**
	**Adequate**	**Inadequate**		
**Sex**				
Male	49	26	2.02(1.02, 3.81)	1.51(0.66, 3.43)
Female	42	45	1.00	1.00
**Age**				
≤ 25	27	11	3.66(1.59, 8.35)	2.30(0.69, 7.70)
26 – 29	31	11	4.19(1.85, 9.48)	2.81(0.99, 7.97)
≥ 30	33	49	1.00	1.00
**Marital status**				
Never married	50	18	3.59(1.83, 7.06)	3.05(1.11, 8.39)*
Ever married	41	53	1.00	1.00
**Monthly income**				
Low	50	18	0.11(0.05, 0.22)	0.26(0.11, 0.65)*
High	41	53	1.00	1.00
**Job satisfaction**				
Very satisfied	17	7	2.80(1.01, 7.81)	1.98(0.59, 6.67)
Satisfied	48	34	1.63(0.82, 3.23)	2.67(1.12, 6.37)*
Unsatisfied	26	30	1.00	1.00
**Field of study**				
MD & HO	22	2	5.92(1.07, 32.90)	3.24(0.20, 51.41)
Nurses	30	50	0.32(0.12, 0.90)	0.52(0.15, 1.80)
Laboratory & pharmacy	26	12	1.17(0.37, 3.67)	0.89(0.23, 3.40)
Others	13	7	1.00	1.00
**Education**				
Greater than degree	20	2	9.72(2.19, 43.16)	0.75(0.05, 11.19)
Degree and diploma	71	69	1.00	1.00
**Computer training**				
Trained	56	22	3.56(1.85, 6.87)	2.81(1.22, 6.43)*
Not	35	49	1.00	1.00
**Owned computer**				
Owned	41	6	8.88(3.50, 22.58)	6.35(2.00, 20.18)*
Not	50	65	1.00	1.00

*MD *Medical doctor, *HO *Health officer and others refers to environmental health, dentistry, radiography and physiotherapy.

* Indicates significant at p <0.05.

**Table 4:** Factors associated with fair knowledge of computer among health workers at Addis Ababa administrative hospitals Ethiopia, June 2010

**Variables**	**Knowledge**		**COR (95% CI)**	**AOR (95% CI)**
	**Fair**	**Inadequate**		
**Age**				
≤25	20	11	1.82(0.79, 4.19)	1.05(0.35, 3.19)
26-29	39	11	3.55(1.63, 7.72)	2.54(1.04, 6.22)*
≥30	49	49	1.00	1.00
**Marital status**				
never married	46	18	2.19(1.13, 4.21)	1.77(0.72, 4.37)
ever married	62	53	1.00	1.00
**Monthly income**				
Low	46	18	0.23(0.11, 0.47)	0.34(0.15, 0.77)*
High	62	53	1.00	1.00
**computer training**				
Trained	56	22	2.40(1.28, 4.50)	1.88(0.92, 3.87)
Not	52	49	1.00	1.00

* Indicates significant at p <0.05.

### Utilization of computer

A total of 38(14.1%) had adequately utilized computer, 14(5.2%) demonstrated average or fair utilization and majority of the respondents 218(80.7%) inadequately utilized computer. Males than female [AOR- 3.06, 95% CI (1.13, 8.27)], those who were satisfied with their job than not satisfied with their job [AOR- 3.41, 95% CI (1.08, 10.76)] and who owned computer than not owned personal computers were more likely to adequately utilize computer. Respondents with less income and nurses were less likely to utilize computers adequately (Table 5). People with less income were also less likely [AOR-0.11, 95% CI (0.02, 0.56)] to fairly utilize computers than high income groups (Table 6).

**Table 5:** Factors associated with adequate computer utilization among health workers at Addis Ababa administrative hospitals Ethiopia, June 2010

**Predictor variable**	**Utilization**			**AOR (95% CI)**
	**Adequate**	**Inadequate**	**COR (95% CI)**	
**Sex**				
Male	26	88	3.201(1.53, 6.68)	3.06(1.13, 8.27)*
Female	12	130	1.00	1.00
**Age**				
≤ 25	5	50	0.54(0.19, 1.52)	2.30(0.69, 7.70)
26 – 29	13	60	1.17(0.54, 2.52)	2.81(0.99, 7.97)
≥ 30	20	108	1.00	1.00
**Monthly income**				
Low	4	159	0.05(0.02, 0.17)	0.18(0.05, 0.71)*
High	34	59	1.00	1.00
**Job satisfaction**				
very satisfied	8	34	2.80(0.94, 8.30)	1.98(0.59, 6.67)
Satisfied	23	101	2.70(1.10, 6.61)	3.41(1.08, 10.76)*
Unsatisfied	7	83	1.00	1.00
**Field of study**				
MD & HO	20	16	4.46(1.54, 12.95)	1.59(0.28, 9.02)
Nurses	5	130	0.137(0.40, 0.47)	0.18(0.42, 0.80)*
laboratory & pharmacy	6	47	0.46(0.14, 1.50)	0.89(0.23, 3.40)
Others	7	25	1.00	1.00
**Level of education**				
Above BSc	17	11	15.23(6.31, 36.78)	0.93(0.18, 4.96)
BSc and below	21	207	1.00	1.00
**owned computer**				
Owned	25	38	9.11(4.28, 19.40)	7.19(2.59, 19.99)*
not	13	180	1.00	1.00

*MD *Medical doctor; *HO *Health officer, & others include environmental health, dentistry, physiotherapy & radiography.

* Indicates significant at p-value <0.05.

**Table 6:** Factors associated with fair computer utilization among health workers at Addis Ababa administrative hospitals Ethiopia, June 2010

**Predictor variables**	**Utilization**		**COR (95% CI)**	**AOR (95% CI)**
	**Fair**	**Inadequate**		
**Age**				
≤ 25	3	50	2.16(0.42, 11.08)	1.70(0.30, 9.74)
26–29	8	60	4.800(1.23, 18.78)	4.09(0.95, 17.65)
≥ 30	3	108	1.00	1.00
**Monthly income**				
Low	5	159	0.206(0.206, 0.640)	0.11(0.02, 0.56)*
High	9	59	1.00	1.00

*MD *Medical doctors, *HO *Health officer, *AOR *Adjusted odds ratio, *CI *Confidence interval.

* Indicates significance at p <0.05.

## Discussion

In this study, knowledge was very low compared to findings in previous studies [9,10]. These may be due to socio-economic differences and diffusion rate of technology among those study subjects. However, findings in this study showed higher knowledge of computer than a study in Nigerian University teaching hospital staffs [6,7]. These differences might be attributed the time gap between the two studies conducted.

Among the socio-demographic variables; marital status, average monthly income and job satisfaction had statistically significant association with adequate computer knowledge. On top of this, computer training and Own Computer possessions were also statistically significant with adequate computer knowledge. On the other hand Age and Average Monthly Income had significant association with fair computer knowledge.

Those never married were more likely to have adequate knowledge on computer than ever married. This is inconsistent with the findings of a previous study from Nigeria [6] which may be due to differences in the study designs. Health workers with less average monthly income were less likely to have adequate knowledge of computer. On the other hand satisfied participants were more likely to have adequate knowledge of computer. These might be associated with the ability to afford the trainings and exposure to computers. On top of this, participants who had had computer training were more likely to had adequate computer knowledge than those who did not train. This is a logical finding that training should bring knowledge change on the trainee side. Similarly participants who had their own computer were more likely to had adequate knowledge than who had not own computer. This is also true or consistently in line with the saying that “innovation can be adopted after having knowledge about the innovation to be adopted”.

On the other hand sex, age, professional variability and levels of education were not statistically significant with adequate computer knowledge of the study participants. This study was consistent with a similar study conducted in Nigeria [6]. But a study in India showed that age had significant association with computer knowledge [10].

Only few of the study participants reported adequate and fair computer utilization in this study. This finding was similar with a study conducted in India [10]. On the other hand, it was very low when compared to other studies carried out in different places [6,13-15]. This could be because of difference in economic status and the rate of diffusion of information technology among the study setups.

In this study sex, average monthly income, job satisfaction and professional background had significant statistical association with computer utilization. Additionally possession of own computer was also statistically significant with computer utilization. Only Average Monthly Income had significant association with both adequate and fair computer utilization.

In this particular study, males were more likely to use computers adequately than females. This might be due to fewer females with personal computers; accessibility of computer may differ in the place of work due to the position held by majority of women. Those health workers with low average monthly income were less likely to adequately utilized computer. Whereas satisfied study participants with their work were more likely to utilize computer than unsatisfied participants and this may be attributed to purchasing power and factors of motivation in the work places.

Regarding the professional background, nurses were less likely to adequately utilized computer. Possible explanation could be due to the work load of nurses compared to their contemporaries. Ownership of computer increases the likelihood of utilizing computer which might be associated with physical accessibility and familiarity with the technology. On the other hand age, marital status, levels of education and computer training were not statistically significant with the computer utilization habit of the study participants. This finding was similar to other studies [6,11]. It may be also similarity of study participants in some of their background characteristics. In this study, actual computer competence and attitude towards computer use was not measured and in some of the categories the sample size was small and this might have resulted in imprecise estimation in the computers utilization measurement. The validity and reliability of the instrument was not checked in this particular population.

## Conclusions

Computer knowledge and utilization habit of health workers were found to be very low. Generally, increasing accessibility of computers and delivering trainings on computers for health workers increases knowledge and utilization of computers or increases the rate of diffusion of the technology for the health sector. Hence, programs targeted at enhancing knowledge and skill of computer use and increasing access to computer should be designed. The ministry of education should enhance computer literacy for health professionals training. The ministry of health needs to design in-service trainings on computer skills. There should be an observational study on the skill of health workers on computer use. The association between computer knowledge/skill and health care delivery competence should be studied with rigorous study designs.

## Competing interests

The authors declare that they have no competing interests.

## Authors’ contributions

EM, GA and SM designed the study, analyzed the data and drafted the manuscript. EG was involved in analysis of the data and critically reviewed the article. All authors read and approved the final manuscript.
